# Smartphone Location Recognition with Unknown Modes in Deep Feature Space

**DOI:** 10.3390/s21144807

**Published:** 2021-07-14

**Authors:** Nati Daniel, Felix Goldberg, Itzik Klein

**Affiliations:** 1 Technion-Israel Institute of Technology, 1st Efron st., Haifa 35254, Israel; 2 Department of Marine Technologies, University of Haifa, 199 Aba Khoushy Ave., Haifa 3498838, Israel; felix.goldberg@gmail.com (F.G.); kitzik@univ.haifa.ac.il (I.K.)

**Keywords:** accelerometers, activity recognition, anomaly detection, deep feature space, machine learning

## Abstract

Smartphone location recognition aims to identify the location of a smartphone on a user in specific actions such as talking or texting. This task is critical for accurate indoor navigation using pedestrian dead reckoning. Usually, for that task, a supervised network is trained on a set of defined user modes (smartphone locations), available during the training process. In such situations, when the user encounters an unknown mode, the classifier will be forced to identify it as one of the original modes it was trained on. Such classification errors will degrade the navigation solution accuracy. A solution to detect unknown modes is based on a probability threshold of existing modes, yet fails to work with the problem setup. Therefore, to identify unknown modes, two end-to-end ML-based approaches are derived utilizing only the smartphone’s accelerometers measurements. Results using six different datasets shows the ability of the proposed approaches to classify unknown smartphone locations with an accuracy of 93.12%. The proposed approaches can be easily applied to any other classification problems containing unknown modes.

## 1. Introduction

Human activity recognition (HAR) is a task aimed to automatically recognize human’s physical activity. HAR is applied in many fields and applications, such as health monitoring, smart homes, sports, security, context awareness and indoor navigation. Starting in the 1990s [1], HAR has emerged as a key problem to ubiquitous computing, human-computer interaction and human behavior analysis with [2,3,4]. There, sensory data obtained from wearable sensors [5] are used to identify a human activity. One possibility for collecting sensor data is by utilizing the channel state information of Wi-Fi signals [6] or by leveraging from multiple wearable devices sensors such as inertial sensors (accelerometers, gyroscopes and barometer) and ambient environment sensors (temperature and humidity) [7,8].

Focusing on indoor navigation, HAR approaches enable the possibility of classifying the user dynamics (mode), for example walking or running. This is made possible by using the smartphone’s inertial sensor. Knowledge of the user mode, helps to improve the positioning accuracy. For example, in [9] statistical measures were employed to distinguish between walking and running user modes. Was classified, a selection of appropriate model parameters was made. Later, four types of user modes, namely, walking, running, bicycle, and vehicle were addressed in [10].

An important branch of HAR in indoor navigation is smartphone location recognition (SLR) [11,12]. It refers to the process of identifying the location of a smartphone on the user during specific actions. For example, the smartphone will be judged to be in Texting mode when the user holds the phone and writes a text massage or in Talking mode when the user hold the phone during a phone call. Identifying those smartphone modes helps to improve pedestrian dead reckoning performance as was shown in [13]. In [14] both user and smartphone modes were addressed including eight types of user modes and seven types of smartphone locations. There, a distinction was made between several types of the Texting operation, namely with one or two hands and additionally in the Swing mode between big or small arm swing. In [15] a finite state machine was used to identify three smartphone locations: Swing, Texting, and Pocket. To assist in the integration of the accelerometers and gyroscopes, a threshold-based approach was used to distinguish between the presence and absence of a Swing mode [16]. Recently, smartphone mode recognition was used to improve heading determination performance [17].

Most of the papers addressing HAR or SLR uses a dataset with specific known modes (for instance: Talking, Texting, or Swing) in a supervised learning approach. When the user encounters a previously unknown mode (for instance, Pocket), the classifier will perforce identify it as one of the original modes it was trained on. Such classification errors will degrade the navigation solution accuracy. This problem is not unique to HAR or SLR, of course, and is known in the machine learning (ML) literature under a number of different names, guises, and variations such as classification with a reject option, one-class classification, anomaly detection, and open set recognition, to name a few. We refer the reader to the recent survey [18] for a more detailed and nuanced exposition on the different variants. Also to [19] for a theoretical analysis.

Consider the following common scenario: a user is walking with a smartphone and a pedestrian dead reckoning (PDR) algorithm to estimate the user position is applied. In such an algorithm, different parameters are used pending on the user dynamics (Walking/Standing/Escalators and etc.) and smartphone locations (Texting/Talking/Pocket and etc.). For example, different user dynamics or smartphone location will result in different PDR gain values or different network parameters for estimating the pedestrian position. Focusing on the smartphone location, SLR classifies it and appropriate PDR parameters are selected. In situations of unknown smartphone locations, that is smartphone locations which are not defined in the PDR algorithm, it is desired to take some average gain value from all defined modes to minimize the positioning errors. Otherwise miss classifying the unknown mode as one of the known modes will result in 10% position error as shown in [13] in a 21 m trajectory. For longer ones, the error is expected to increase. Thus, a methodology to cope with unknown modes is needed to be incorporated in the PDR algorithm.

To fill this gap, in this paper we propose two end-to-end ML-based approaches to address the unknown smartphone location modes problem using only the smartphone’s accelerometers measurements: (1) a supervised approach which requires the known modes labelling during the training phase and (2) an unsupervised approach without the requirement to label training data. In both approaches, a feature representation space is extracted and fed into a K-nearest neighbors algorithm to detect the unknown modes.

To enhance the efficiency and robustness of the proposed approaches multiple datasets are used in the training and testing phases. The training process is based on four different datasets recorded by 23 people while the smartphone was placed in four different known smartphone locations: Texting, Pocket, Swing, and Talking. The test dataset contains two additional datasets recorded by 25 people (not present in the train dataset) with the following five smartphone locations treated as unknown modes: Body, Bag, Belt, Waist, and Upper-arm.

Although our focus is unknown modes in the SLR problem, the proposed approaches can be easily adjusted to any other domains requiring unknown modes classification.

The rest of the paper is organized as follows: Section 2 reviews current approaches to handle unknown modes while Section 3 presents the mathematical foundations required for the proposed approaches. In Section 4, the proposed approaches are described while the datasets used to evaluate them are described in Section 5. Section 6 brings the experimental results of this research. Finally, Section 7 gives the conclusions of this work.

## 2. Related Work

A number of possible existing approaches to handle the unknown mode problem are briefly described. They can be categorized into three groups: (1) Thresholding, (2) Reject option, and (3) Training with a background class.

### 2.1. Thresholding

One possible way to respond to the challenge of unknown modes is by thresholding the classification probabilities of the model that has been trained on the known modes. Usually these probabilities are available as the output of a final softmax activation function in the model. If none of the known modes is recognized with high enough probability, the input is declared as “unknown”. See e.g., [20,21]. More sophisticated manipulations on the vector of classification probabilities are also possible and we refer the reader to [22,23,24] and the references therein.

However, while methods from this class do sometimes work well in practice, their principal shortcoming is that they do not really detect unknown modes at all, but rather produce an estimate of the *confidence* of the model in its selection of one of its known modes. Low confidence is then taken to be an indication of the presence of an unknown mode or anomaly. But since low confidence may have other causes as well, this assumption is rather shaky. And indeed, in many practical scenarios, given an input of an unknown class, the classifier will still pick one of the available modes with high probability, indicating that the threshold approach is not robust enough.

### 2.2. Reject Option

Another possible approach to the unknown mode problem is to integrate the possibility of rejection into the classifier, instead of post-processing its probability estimates [25,26]. One shortcoming of this kind of approach is that it necessitates a rather more complex training regimen. Another, and perhaps more severe, will occur in the situation when a hitherto unknown mode is converted into a known one. This will require a complete retraining of the model.

### 2.3. Training with a Background Class

Another possible type of approach can be taken, when there is ample data belonging to the unknown modes during training. In such scenarios, one can divide the task into two stages; in the first stage a binary classifier is trained to distinguish between known and unknown modes on the basis of the training data and then a second classifier finds the specific mode only for those detected as known in the first stage. This approach is not quite recommended because of its apparent brittleness. In particular, no guarantees can be made at all for its behaviour in the case that a *new* unknown mode is introduced. We refer to [27] for a more detailed critique.

A sturdier version is sometimes used in practical work, wherein all the unknown modes are lumped together as a “background” or “garbage” additional class and the classifier is trained with n+1 labels for *n* known modes. While there is still no theoretical justification for this kind of a solution, it can be intuitively defended on the grounds that the classifier implicitly learns features that distinguish the collection of unknown modes from the known ones and so if we take care to include many different unknown modes in our data collection, it will have a fighting chance to learn useful features.

## 3. Algorithmic Building Blocks

In this section, three commonly used algorithmic building blocks are briefly reviewed. Those, later, will be employed as part of the proposed approaches as presented in Section 4.

### 3.1. K-Nearest Neighbors

K-Nearest Neighbors algorithm (KNN) is an elementary supervised ML algorithm for easy interpretation, versatile and low calculation time of both classification, regression predictive problems or anomaly detection [28]. KNN is a non-parametric method for instance-based leaning. In other words, there are no assumptions on the underlying data distribution, and it does not need any training data points for mathematical model generation. In general, the KNN output depends on the application, for instance, in classification (a class membership), in regression (a property value) and in anomaly detection [29] (outlier score). There, *K* is the core design parameter, which indicates the number of nearest neighbors, and depends on the model building procedure.

To formulate this algorithm, consider a train dataset arranged as a set of *n*-pairs:

(1) $$\left(X_{1},Y_{1}\right),\left(X_{2},Y_{2}\right),\ldots ,\left(X_{n},Y_{n}\right)$$

where $Y_{i}$ is the class target of $X_{i}$ features vectors for i=1,2,3,…,n. So that X|Y=r for r=1,2 (e.g., two classification classes). Given some norm ${\left||\cdot |\right|}^{2}$ on $R^{d}$ and let $q\in R^{d}$ be a query point for which target needs to be predicted. Hence, the first *K* closest points from the given train dataset to *q* should be found by calculating the distance between the train dataset points to *q*. Commonly, Euclidean distance Equation (2) is applied followed by a second vote for target by either a majority vote of most common among its *K* nearest neighbors or the average of the values of *K* nearest neighbors.

(2) $$d\left(X,q\right)={\left||X-q|\right|}_{2}$$

### 3.2. Principal Component Analysis

Principal Component Analysis (PCA) is one of the most widely used unsupervised dimensionality reduction techniques for data exploration, statistical analysis and predictive modelling [30,31]. During the PCA process, the principal components are computed and used to transform the data into a subspace, without relying on class labels. In other words, PCA has no concern with the class labels. In general, it returns a compact representation of a multidimensional dataset by reducing the dataset to a lower dimensional subspace. In particular, these principal components are based on transforming a large set of variables into a small set of *p*-variables, which are linearly uncorrelated, by finding orthogonal linear combinations of the original variables with largest variance. As a result, the first principal component has the highest variance, the second principal component has the second highest variance, and also it is orthogonal to the first principal component, and so on. Often, only the firsts principal components consist of most of the variance in the original dataset, and therefore, in many applications only they are used while the rest principal components, with minimal loss of the variance, are ignored. From either objective, it can be shown that all of the variation in the original dataset is accounted for by the principal components.

### 3.3. Linear Discriminant Analysis

Linear Discriminant Analysis (LDA) is a supervised dimensionality reduction technique of data for easy exploration, statistical analysis and predictive models [32]. It is the process of reducing the number of dimensions (i.e., variables) in the dataset while retaining the information that discriminates output classes. That is, LDA tries to find a decision boundary around each cluster of a class labels. In general, [33] is a generalization of Fisher’s linear discriminant, which seeks an optimal set of discriminant projector vectors to map a multidimensional dataset onto a lower dimensional subspace. In particular, these linear discriminants are based on the projection of the dataset in a way that the classes are as separate from each other as possible and the individual elements within a class are as close to the mean of the class as possible. Then, the new dimensions are ranked on the basis of their ability to maximize the distance between the classes (i.e., the fisher criterion) and minimize the distance within the classes [34]. In this way, the ratio of the projected dataset between to within class scatter is maximized.

## 4. Proposed Ml-Based Approaches: Anomaly Detection in Deep Feature Space

**Figure 1:**
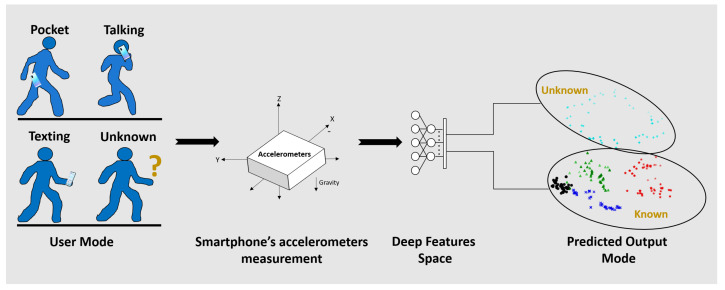
Proposed approach: accelerometer readings are represented in deep feature space to classify unknown smartphone modes.

The goal of this paper is to provide an effective mechanism for the detection of accelerometer signals belonging to unknown modes. To that end, the SLR domain is chosen to derive and present the proposed approach, but the it can be easily adapted in any other activity recognition tasks and in other domains as well. It is assumed that a separate model capable of classifying the known SLR modes exists, for example, as in [11]. There the four known smartphone locations (classes) are: (1) Texting, (2) Swing, (3) Pocket, and (4) Talking. The central idea of our solution is as follows: a high-quality classification model for the known modes will have already learnt a good deep feature representation of the accelerometers readings. This representation is essentially a function $f:R^{n}\to R^{d}$, where *n* is the input dimension of the signal (n=3 in our case) and *d* is the dimension of the hidden representation. The proposed approach is illustrated in Figure 1.

Two different approaches to utilize the feature representation in order to classify unknown modes are suggested:

1. **SUN: supervised unknown network:** The penultimate layer of a network, trained in a supervised manner from data belonging to a number of known modes, is employed for the feature representation space. Then, a KNN algorithm is applied to determine if the signal belongs to a known or an unknown mode. Another possibility is to apply standard techniques of dimension reduction (PCA or LDA) prior to KNN application.
2. **UUN: unsupervised unknown network:** Novel features extracted from a latent representation using a variational recurrent auto-encoder (VRAE) architecture, trained in an unsupervised manner, are used as the feature representation space. Then, a KNN algorithm is applied to determine if the signal belongs to a known or unknown modes. Unlike in the SUN approach, here the labeling of the known modes are not needed in the training process.

**Figure 2:**
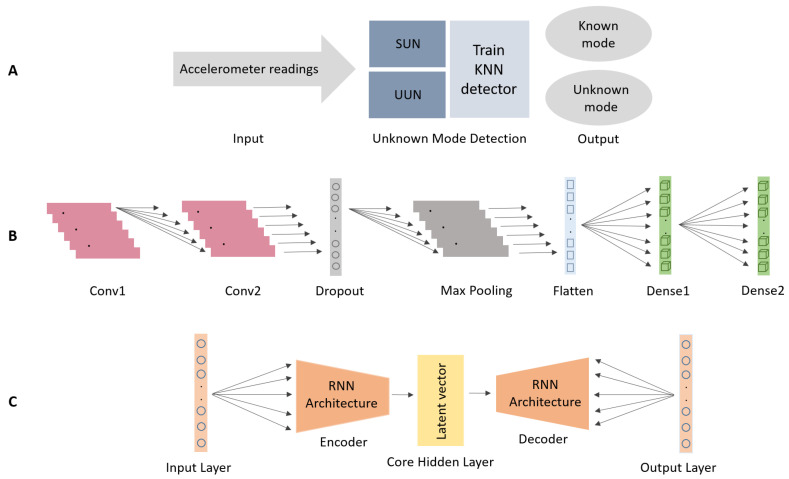
Proposed approaches for unknown modes detection using the smartphone’s accelerometers. (**A**) SUN and UNN with a KNN for separately modelling the unknown modes networks, (**B**) SUN network architecture used in training, and (**C**) UUN network architecture used in training.

With the representation in hand (either by SUN or UUN approaches), a robust and effective way to detect unknown modes is via *anomaly detection* in the representation space. To that end, the non-parametric method of [29], as implemented in the PyOD toolbox [35], will be employed. In other words, we will apply the KNN algorithm of [29] to *d*-dimensional feature vectors representing a signal instance and obtain either a TRUE or FALSE answer. TRUE means that we have on our hand an anomaly, which means that the signal is from an unknown mode, while FALSE means that the signal belongs to a known mode. The SUN and UUN approaches are shown in Figure 2A.

Now after the proposed approaches are defined, they are compared to Thresholding, Reject option, and Training with a background class approaches (as described in Section 2) in Table 1. Notice, that for new unknown mode addition and unknown-known conversion both SUN and UUN proposed approaches don’t require additional retraining procedure.

**Remark** **1.** *The emphasis we place on the role of the deep feature representation as opposed to more sophisticated rejection mechanisms is inspired by the pervasive role deep features play in visual image understanding where it is by now well-established that good features allow strong generalization across tasks (cf., e.g., [36,37]), which is exactly what we seek here. This also allows for smooth handling of the appearance of new unknown modes since they usually do not affect the feature representation*

### 4.1. SUN Network Architecture

Motivated by [11], a supervised one-dimensional convolution neural network (1D-CNN) model was trained on the four known modes. The CNN architecture that is used for training the different SLR modes is shown in Figure 2B. The input to the network is the accelerometer measurements (specific force vector). The first layer, is a 1D-CNN with 32 units and a ReLU activation. The second layer is identical to the first one. After a dropout of 0.6, the next layer is a 1D-Polling layer of size two followed by a flatten layer to set the dimensions to the following two dense layers. The dense layer has 32 units with a ReLU activation function and the final layer (dense) has a Softmax activation to output the SLR classification result. That is, given the accelerometers readings the network outputs one of the four known modes.

### 4.2. SUN Training Procedure

The network is trained with a minibatch of size 32, and the RMS propagation (RMSProp) [38] algorithm for optimization, which divides the gradient by a running average of its recent magAdam optimizer, is used. An initial learning rate of λ=0.001, a discounting factor for the history/coming gradient of ρ−0.9, and zero momentum are applied. Dropout is applied after the convolutional neural network (CNN) layers, with probability 0.6. The network is trained for four different SLR modes (Swing, Texting, Talking, Pocket), using the categorical cross entropy (CCE) loss function defined in [39] for a single label categorization. The network is trained for 12 epochs. This model was implemented using Keras open-source neural network library in Python [40] and was trained on a single NVIDIA GeForce GTX 1080 GPU.

### 4.3. UUN Network Architecture

The UUN approach uses the VRAE [41] network architecture. This model maps time sequences to one latent vector, and enables efficient, large scale unsupervised variational learning on time sequences, while it tries to avoid the exploding gradients problem and enable better scores. The main concept behind it is to partition unlabeled time-series measurements into homogeneous clusters based on generative features, which are interpretable.

The strength of VRAE architecture is that it extends the standard variational auto-encoder (VAE) model by combination of recurrent neural networks (RNNs) as the network encoder and decoder, and stochastic gradient variational Bayes (SGVB) [42]. The VRAE architecture that is used for training the different SLR modes is presented in Figure 2C. It receives the specific force vector measurements as input to the encoder RNN layer, which contains enrolled long short term memory (LSTM) block, with hidden size of 90, depth of 3, and dropout rate of 0.3. Then, the RNN output is passed to the next, encoder to latent, layer which is mapped to the mean and standard deviation by using a liner unit activation. When the hidden code layer size of 20 dimensions, and distributed by mean and standard deviation that serves as the feature representation for the entire input during training (i.e., encodings). Next, the latent layer is passed through a linear unit activation to obtain initial states for the decoder RNN layer. Decoder inputs are updated using backpropagation.

The UUN model was trained to minimize smartphone known modes loss function for accelerometer readings *X*, defined by:

(3) $$Loss\left(X\right)=C_{SL}\cdot Smooth_{L1}\left(X\right)+C_{KL}\cdot D_{KL}\left(X\right)$$

where the loss function is a superposition of two loss measures—Smooth L1 and Kullback-Leibler (KL) divergence function, each with a corresponding gain $C_{SL}$, and $C_{KL}$, receptively.

The $Smooth_{L1}\left(X\right)$ loss measure is an auto-encorder loss that learns the identity function, so the sequence of input and output vectors must be similar. It is given by:

(4) $$Smooth_{L1}\left(X\right)=\frac{1}{n}\sum_{i}^{n}z_{i}$$

where $z_{i}$ is defined by:

(5) $$z_{i}=\left\{\begin{array}{cc} 0.5{\left(x_{i}-y_{i}\right)}^{2}, & \mathrm{if}\;|x_{i}-y_{i}|<1\\ |x_{i}-y_{i}|-0.5, & \mathrm{otherwise}\; \end{array}\right.$$

and *x* (input) and *y* (target) are in arbitrary shapes with a total of *n* elements each. This loss was shown to be less sensitive to outliers than the mean square error loss and in some cases prevents exploding gradients [43].

The second part of the loss function Equation (3), is the KL-divergence function, a loss measure between the distribution that learned in latent space with the normal distribution, defined by:

(6) $$D_{KL}\left(X\right)=-\frac{1}{2}\sum_{j=1}^{L}\left(1+log\left({\left(\sigma_{j}^{i}\right)}^{2}\right)-{\left(\mu_{j}^{i}\right)}^{2}-{\left(\sigma_{j}^{i}\right)}^{2}\right)$$

where *L* be the latent continues variable length, μ the variational mean, and σ is the standard deviation evaluated at datapoint $x_{i}\in X$.

### 4.4. UUN Training Procedure

The network is trained with a minibatch of size 32 and the Adam optimizer, with $\beta_{1}=0.9$, $\beta_{2}=0.999$ and $\epsilon =10^{-8}$. We use an initial learning rate of λ=0.0005 and a $L_{2}$ penalty of 0 for the training process. Dropout is applied in the encoder layer (that uses a multi-layer LSTM) with probability 0.2. The network is trained for four different SLR modes (Swing, Texting, Talking, Pocket), using the loss function defined in 3. Notice, that the modes labels are not used during the training process. The train is made with $C_{KL}=-1$; $C_{SL}=1$, when in such a case, the weight for both losses is equal for learning. The network is initialized with Glorot and Bengio [44], which proposed to adopt a properly scaled uniform distribution for initialization, and train it for 90 epochs, with Gradient clipping enabled and max norm of the gradients of size 5, to overcome explosion. The model was implemented using PyTorch [45] and trained on a single NVIDIA GeForce GTX 1080 GPU.

## 5. Dataset

To evaluate the proposed SLR with unknown modes approaches, six different datasets were used. Two of the datasets were constructed for evaluation deep-learning approaches in the SLR problem [11], while the other four datasets, found in the internet were constructed for other applications. In all the datasets, the smartphone location was, at least, in one of the four possibilities: (1) pocket, (2) texting, (3) swing, and (4) talking while the users were walking. No constraints on how the smartphone should be held in each location was imposed. For example, talking operation can be made while the user is holding the phone in the right hand close to the ear or in the left hand far from it. In all recordings only the accelerometer readings are used for the proposed approaches.

For the training process four different datasets, as described in Table 2, were employed.

For the SUN approach, the known labels were used during the training while for the UUN they were not. The first dataset, D1, contains recordings from a single user with two different smartphones and in all four possible smartphone locations. A total of 164 min of recordings were made using a range of sampling rates between 25 and 100 Hz. The recorded data was made while the user was walking in inhomogeneous conditions. For example, varying walking speeds, uneven pavements, tight and sport trousers with a front and back pocket location, transitions between pavement and roads, varying hand swing (small to big), and texting and talking with a single hand (right and left) in different positions relative to the user.

The second dataset, HTA, was recorded by six people from Huawei’s Tel-Aviv research center. Each person used a different smartphone during the recordings. The third dataset used in this research is RIDI [46]. This dataset was recorded for indoor navigation research and not activity recognition. There, the goal was to estimate the change in acceleration by machine learning approaches and use it to correct the raw accelerometer data. Although, their work was not related to SLR, the RIDI dataset was recorded using a smartphone in two locations: front pocket and in texting using eight people. The fourth dataset, OXF, was recorded to examine the possibility of using deep learning methods to estimate the pedestrian position and heading [47]. There, a dataset using seven people with a time duration of 240 min was recorded while the smartphone was in Pocket or Texting modes were made.

The test dataset contains five smartphone locations, not present in the training data, which are addressed as unknown mode. Those are: Waist, Upper-arm, Belt, Body, and Bag. From RIDI [46] the Body and Bag mode was taken for the analysis. There, the smartphone was placed in a small bag that was hold on the right leg and with a strap on the body. In [48], an HAR problem using wearable sensors located in seven positions on the user: chest, forearm, head, shin, thigh, upper arm, and waist was addressed. This dataset, noted as WOB, contains recordings of 15 people (seven females and eight men) and here we employ only recordings taken from the Waist and Upper-arm location as an unknown mode. The Third one, [49], also addressed an HAR problem with seven different modes among them was walking. There, five smartphones were placed on each user: right/left jeans pocket, right upper arm, wrist and on the belt. There, the smartphone was pointed towards the right leg using a belt clip. Here, we employ the latter as and the Body an unknown modes, and denote this dataset as PAR. Main parameters of those three datasets are given in Table 3.

## 6. Analysis and Results

### 6.1. Why a Regular Supervised Approach Does Not Work?

There are two main reasons that explain why regular supervised networks will fail and are not enough accurate nor robust methods, with presence of unknown modes:

1. **Catastrophic interference:** Deep learning networks, especially supervised architectures, suffer from the catastrophic interference, which is the tendency of the networks to forget previously learned classes upon learning new classes that added incrementally during the training procedure. Thus, it causes a significant degradation issue when creating connectionist models, in particular, with deep architectures which aims to solve both vision and real-time smartphone application problems. Therefore, it does not work with presence of unknown modes.
2. **Class imbalance:** Class imbalance is a common problem in deep learning classification predictive models, where the distribution of samples across the known classes is biased from the other possible different classes, when in most of the cases are much larger. Thus, the class imbalance phenomena will make a significant degradation in the network performance, specially for the targeted minority class. Therefore, it also does not work accurately with presence of unknown modes.

To confirm this hypothesis, a traditional CNN supervised model was trained on five SLR modes: Pocket, Swing, Texting, Talking, and Unknown. To that end, the dataset listed in Table 2 was used as train with the addition of most of WOB and PAR datasets provided in Table 3 as unknown modes. The rest of WOB and PAR dataset was addressed as similar test dataset while RIDI dataset, also given in Table 3, was addressed as dissimilar test dataset.

**Figure 3:**
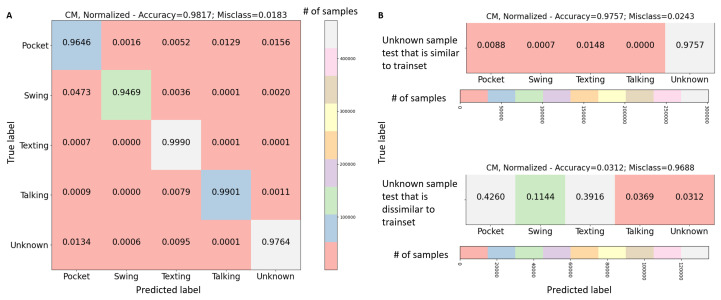
Results for regular supervised network. (**A**) Training five different SLR known modes with supervised CNN architecture, ((**B**) top) Testing unknown data with similar dynamics, achieves high accuracy, ((**B**) bottom) Testing unknown data with dissimilar dynamics, achieves very low accuracy.

The train model accuracy (based on F1-Score) obtained 98% as shown in Figure 3A. As expected, given the unknown data as another class in the training can be easily handled by the network. The similar test dataset obtained also high accuracy of 95.2% as shown in Figure 3B. There, the same unknown location was used only with different people. However, for the dissimilar test dataset, an accuracy of 3.11%, as presented in Figure 3B bottom, was observed. To summarize, in such approach if the unknown modes are actually known in the training process then they should be addressed as any other known classes. Yet, given unknown modes not present in the train, this approach fails and other solutions are required.

### 6.2. SUN and UUN Approaches

Utilizing the SUN approach, the last dense layer of the the supervised trained model is truncated and used as the feature representation space. To that end, training is applied as described in Section 4.2. Using the trained network three methods are considered based on the SUN approach:

- **SUN-RAW:** the supervised raw features are directly used in the KNN algorithm, using K=5 nearest neighbors to classify unknown modes (5 seems to be the most commonly used value for KNN algorithm, other experiments showed that higher K values increase the error rate).
- **SUN-PCA:** as in SUN-RAW only with PCA mapping of the raw features to a lower-dimensional space is made prior insertion to the KNN algorithm (K=5). After some analysis, the number of principal components are set to two.
- **SUN-LDA:** as in SUN-RAW only with LDA mapping of the raw features to a lower-dimensional space is made prior insertion to the KNN algorithm (K=5). After some analysis, the number of discriminant components set to three.

**Figure 4:**
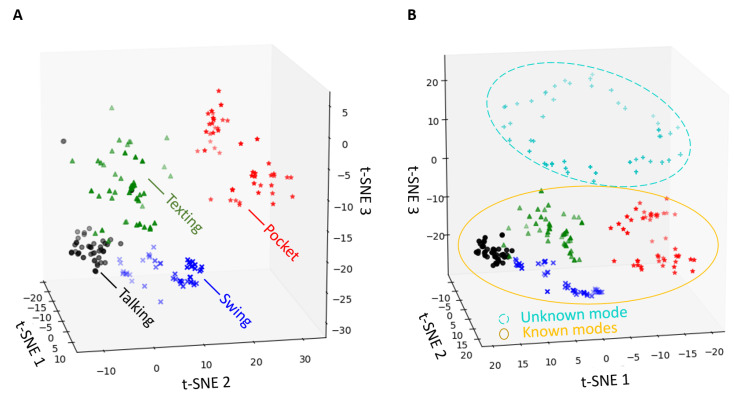
UUN feature representation. (**A**) Training representation, (**B**) Inference representation. t-SNE is performed in order to convert to a lower dimension and to visualize the clusters. This figure shows that latent space exhibits structure. The different colored labels obviously cluster in different parts of the UUN feature space. t-SNE axes 1, 2, and 3 represent dimension 1, 2, and 3, respectively.

The UUN network is trained as presented in Section 4.4. There, the unsupervised features representation space is directly plugged to the KNN algorithm, using K=5 nearest neighbors to classify unknown modes. Illustration of the feature space representation in UUN approach is shown in Figure 4 both for the training phase and the inference phase of inserting an unknown mode. The representation of the unknown mode is clearly seen. Note, that the entire algorithm is an unsupervised one. Labels are used to color and visualize the results.

The parameters setting of the PCA and LDA methods utilized in the SUN approach are presented in Table 4, and the KNN setting applied in both SUN and UUN approaches is given in Table 5. Notice, before determining the PCA number of components, different values ranging from two to ten were examined. When the number of components was two, best performance was obtained and therefore it was chosen for further analysis. In the same manner, the number of LDA components was determined.

Table 6 shows the performance of the proposed approaches presenting the accuracy of each method in six different unknown modes from three datasets. Among them, PAR and WOB were not present at all in the training dataset while RIDI had some Pocket and Texting modes readings also in the train dataset. In addition, the results are compared to the baseline method of [21] using a threshold of 95%. This baseline belongs to the Thresholding approaches, and is well-established in the literature. The threshold value of 95% was tuned manually for optimal performance of the baseline and to challenge the proposed approaches. If a lower value was chosen the results will be worsen. To summarize, this baseline method achieved low performance with the overall accuracy of 7.5% computed on the six unknown different test dataset.

From all three SUN network methods, SUN-RAW obtained the best performance in each of the examined six unknown modes. It appeasers that the dimension reduction using either PCA or LDA degraded a lot the performance. Also, PCA and LDA were found to be highly sensitive to the number of components, where in some situations each unknown mode needs a different number of components to obtain the best performance. Nevertheless, regardless the number of components SUN-RAW was preferable.

In addition to the results, the proposed approaches were compared on the inference average time over the different datasets as shown in Table 7. It was shown that the dimension reduction approaches (PCA and LDA), as expected, managed to reduce by approximately 20 times lower than SUN-RAW and 10 times than UUN approaches.

Table 8 shows the overall accuracy computed on the six different test dataset. SUN-RAW shows the best performance with 93.12% of accuracy while UUN obtained 88.85%. UUN approach results are ranked at the second place mainly due to the performance achieved on the RIDI Body dataset. However, the big advantage using UUN is that there is no need to pre-label the training data, which makes it easier to use such an approach in further research studies or other applications in which the raw data labels are not known.

In addition to accuracy, the training time of SUN and UUN are compared using a mini-batch of size 32. It took five epochs to train the SUN network while in UUN it was 90 epochs which increased the training time by factor of 18. To summarize, SUN-RAW obtained the best performance, yet required much more inference time. On the other hand, SUN-PCA dramatically reduced the inference time, with a cost of approximately 10% of accuracy. Thus, there is a trade-off between accuracy and the computational cost, which should be determined based on the required application.

## 7. Conclusions

SLR aims to identify the location of a smartphone in specific user actions. This task is critical for accurate indoor navigation using PDR. Common PDR approaches cannot handle unknown modes with desired accuracy, and therefore, their performance is degraded. In this paper, two end-to-end ML-based approaches to cope with unknown modes during the classification process were suggested and evaluated on the smartphone location recognition problem.

The first approach, SUN, used a feature representation space of a trained network as its basis, while the second approach, UUN, generated the feature representation space using variational recurrent auto-encoder. Both approaches require only the smartphone’s acceleroemeters measurements to preform the classification and unknown detection.

Multiple datasets were used in the training and testing phases. In training, four different datasets were used. They were recorded by 23 people while the smartphone was placed in four different known smartphone locations: Texting, Pocket, Swing, and Talking. The test dataset contained two additional datasets recorded by 25 people (not present in the train dataset) with the following five smartphone locations treated as unknown modes: Body, Bag, Belt, Waist, and Upper-arm.

Before examining the proposed approaches, it was explained and shown why a classification approach based on background class cannot handle unknown modes effectively in the SLR problem. Then, the performance of a baseline thresholding approach [21] and of the proposed approaches was evaluated The thresholding approach was chosen, since in the literature it was shown to obtain good performance. Yet, in the SLR problem it failed to work and orbited poor accuracy. On the other hand, the proposed SUN achieved an accuracy of 93.12% while UNN obtained 88.85% on the test dataset. The main advantage of the UNN approach is that it does not require labeling the known modes in the training process, however, its training time is 18 times longer than the SUN approach. This is attributed to the computational complexity of the proposed model, which can be evaluated based on the number of total trainable model parameters, where in SUN approach has 17,924 compared to UUN approach with 335,563 parameters. It was also shown that applying PCA or LDA in the SUN approach did not improve its performance, however they reduced the inference time by a factor of approximately 20. Thus, there is trade-off between accuracy and the computational cost, which should be determined based on the required application.

Finally, the proposed approaches were derived as end-to-end ML approaches and thus can be easily adjusted and applied to other related fields addressing the problem of unknown mode detection.

Future work includes an analysis of encoding length of the UUN approach and its influence on the accuracy and the computational cost over different datasets. Additionally, different detectors can be examined, as well as different deep architectures for detecting Unknown modes.

## Figures and Tables

**Table 1:** Comparison between approaches in terms of training data.

Algorithm/Approach	Thresholding	Reject Option	Background Class	SUN	UUN
Requires training data?	No	Yes	Yes	No	Yes
New unknown mode addition requires retraining?	No	Yes	No	No	No
Unknown → Known conversion for a mode requires retraining?	Yes	Yes	Yes	No	No

**Table 2:** Main parameters of all four datasets used in the training process. Smartphone locations are swing (S), texting (T), talking (K), and pocket (P).

Parameter/Dataset	S1 [11]	HTA [11]	RIDI [46]	OXF [47]
# of samples	590,927	43,447	667,099	1,437,622
Total time [Min]	164	15	56	240
# of participants	1	6	8	8
Sampling rate [Hz]	25–100	25–100	200	100
Smartphone modes	S/T/K/P	S/T/K/P	P/T	P/T
Year	2019	2019	2018	2018

**Table 3:** Main parameters of the three datasets used in the testing process.

Parameter/Dataset	RIDI [46]	WOB [48]	PAR [49]
Number of samples	315,725	932,369	167,564
Total time [Min]	26	310	56
Number of participates	8	15	10
Sampling rate [Hz]	200	50	50
Smartphone modes	Bag/Body	Waist/Upper-arm	Belt/Body
Year	2018	2016	2014

**Table 4:** PCA and LDA setting parameters utilized in the SUN approach.

Parameter/Method	PCA	LDA
Number of components	2	3
Tolerance for singular values	$e^{-6}$	$e^{-4}$
Solver	SVD	SVD

**Table 5:** KNN setting parameters utilized in both SUN and UUN approaches.

Parameter/Method	KNN
Number of neighbors	5
Leaf size	30
Radius	1.0
Contamination	0.1
P (distance function)	2
Distance Metric function	Minkowski
Metric to calculate the outlier score	Largest
Algorithm (ball tree/kd tree/brute)	auto

**Table 6:** Accuracy/Number of corrected samples results on different testing datasets used our SUN and UUN approaches.

Method/Dataset	WOB Unknown Waist	WOB Unknown Upperarm	PAR Unknown Belt	PAR Unknown Body	RIDI Unknown Bag	RIDI Unknown Body
Baseline [21]	7.81%/37,687	7.38%/35,633	0.01%/17	2.56%/2259	10.14%/11,694	5.96%/17,345
SUN-RAW *	100%/482,845	92.73%/447,726	99.96%/87,935	100%/87,968	84.12%/20,798	73.71%/214,285
SUN-PCA	99.79%/481,814	20.94%/101,076	95.91%/84,369	99.99%/87,960	72.55%/17,936	33.12%/96,290
SUN-LDA	95.14%/459,423	45.53%/219,816	97.32%/85,617	96.71%/85,704	67.01%/16,568	42.56%/123,718
UUN	99.91%/482,814	82.65%/399,006	99.98%/87,953	99.97%/87,946	85.71%/21,173	62.85%/182,721

* indicates the highest score for the given datasets.

**Table 7:** Inference average time [sec] on different testing datasets.

Method/Dataset	WOB	PAR	RIDI
SUN-RAW	2185.1	2093.3	2344.7
SUN-PCA	106.4	22.4	64.2
SUN-LDA	112.2	27.5	69.7
UUN	1263.9	495.6	956.9

**Table 8:** Summary of Unknown mode ML-based approaches results.

Method	Overall Accuracy
SUN-RAW	93.12%
SUN-PCA	82.31%
SUN-LDA	77.42%
UUN	88.85%
